# Recent Advances in Intelligent Point Cloud Processing, Sensing, and Comprehension

**DOI:** 10.3390/s26103227

**Published:** 2026-05-20

**Authors:** Miaohui Wang

**Affiliations:** Guangdong Key Laboratory of Intelligent Information Processing, College of Electronics and Information Engineering, Shenzhen University, Shenzhen 518052, China; wang.miaohui@gmail.com

## 1. Introduction

Point clouds have become some of the most important 3D data representations in recent years [1]. Because they can accurately describe geometric structures and spatial relationships in practical environments, point clouds are widely adopted in 3D vision-based applications [2], such as autonomous driving, humanoid robotics, intelligent transportation systems, digital twins, remote sensing, industrial automation, and virtual reality. With the rapid development of advanced sensing technologies [3], including LiDAR, RGB-D cameras, structured-light sensors, photogrammetry, and multi-view reconstruction systems, point cloud acquisition is becoming increasingly accessible across a broad range of research and industrial applications.

Recent advances in artificial intelligence, computer vision, machine learning, and geometric data processing have significantly accelerated the development of intelligent point cloud processing, sensing, and comprehension [4]. For example, deep-learning-based techniques [5], geometric feature learning architectures [6], registration algorithms [7], semantic segmentation frameworks [8], and multimodal fusion strategies [9] have greatly improved the robustness and efficiency of point cloud processing systems [10]. However, point clouds remain inherently irregular, unordered, and highly sparse [11], posing considerable challenges for reliable semantic comprehension, geometric reconstruction, and real-time perception in complex real-world environments.

This Special Issue provides researchers and practitioners with a dedicated medium with which to share recent theoretical advances, algorithmic developments, and practical applications related to intelligent point cloud processing, sensing, and comprehension. The accepted publications cover a broad range of hot topics in regard to point clouds, including semantic segmentation, representation learning, registration, geometric analysis, multimodal sensing, intelligent construction monitoring, robotics, industrial safety, and photorealistic scene reconstruction. In summary, these studies demonstrate the increasing importance of point cloud technologies in modern intelligent systems.

## 2. Overview of Contributions

After a rigorous peer-review process, 12 high-quality papers were accepted for publication. They present recent developments in point cloud processing, sensing, and comprehension as well as future research directions.

Specifically, two studies focus on semantic understanding and feature learning. In Contribution #01, a spatial feature structure generation network (SFSGNet) was developed for airborne LiDAR semantic segmentation. By deeply integrating attribute and spatial coordinate features, the proposed spatial feature structure generation module can effectively model semantic and geometric structures within irregular point clouds. Experimental results derived from the ISPRS, DALES, and OpenGF datasets demonstrated promising segmentation accuracy and generalization performance. In Contribution #02, an improved point cloud representation framework based on a learnable sort–mix–attend (SMA) mechanism is introduced. The proposed differentiable module learns task-driven canonical structures for local point sets, enabling efficient MLP-based architectures to capture richer feature interactions. The SMA framework significantly improved the performance of PointNeXt-S and PointNet++ on classification and segmentation benchmarks while maintaining computational efficiency.

Real-time semantic perception and multimodal learning are also important research directions. Contribution #03 presents a real-time semantic segmentation framework for LiDAR point clouds based on multi-view and cross-modal compact feature distillation. The proposed two-branch knowledge-distillation strategy effectively transferred multimodal and multi-view priors into a lightweight LiDAR-only segmentation framework while maintaining real-time performance. Experimental results obtained on SemanticKITTI and nuScenes validated its segmentation accuracy and practical deployment capacity for handheld scanning setups.

Geometric analysis and structural comprehension of point clouds are discussed in Contribution #04, in which a supervoxel-based plane segmentation framework for sensor-acquired 3D point clouds is proposed. By combining boundary adjacency, normal coherence, and projection-line fitting constraints, the proposed method achieved robust segmentation performance in challenging stepwise and intersecting non-coplanar structures. The experimental results demonstrated strong robustness and applicability in practical sensing scenarios.

Furthermore, photorealistic scene reconstruction and intelligent geometric representation are investigated in Contribution #05, in which an enhanced 3D Gaussian splatting (3DGS) framework for real-scene reconstruction is proposed. By integrating depth-aware regularization, adaptive densification, and artifact denoising strategies, the proposed 3DGS method achieved high-quality reconstruction and real-time rendering performance across diverse complex environments. The study further analyzes the trade-offs between depth priors, rendering quality, and computational efficiency, providing valuable insights for scalable, real-world deployment.

Two studies investigate the practical applications of point cloud sensing in building information modeling, industrial automation, and safety monitoring. The authors of Contribution #06 introduce a building-equipment detection method for mobile laser-scanning point clouds using reflection-intensity correction. By compensating for distance and incidence-angle effects, the proposed method effectively extracts building equipment from ceilings and walls, facilitating the automatic generation of detailed building information models (BIMs). Contribution #07 addresses pedestrian intrusion detection in coal-yard hazard areas using 3D LiDAR-based deep learning. The proposed EFT-RCNN framework improves pedestrian detection accuracy and robustness under challenging environmental conditions while enabling real-time operation for industrial safety monitoring.

Moreover, robotic perception and construction automation are further explored in Contributions #08 and #12. Specifically, The authors of Contribution #08 propose a robust pose-estimation and size-classification method for unknown dump trucks using the normal distribution transform, which achieves accurate pose estimation and reliable size classification under various real-world conditions, supporting automated soil-loading operations in construction environments. Contribution #12 presents a robust human-tracking framework for human-following robots using laser range finders. By selecting an optimal projection range from the upper portion of the human body, it significantly improves tracking robustness for people of different heights.

Point cloud registration remains another important topic in intelligent 3D perception. The authors of Contribution #09 propose an improved iterative closest point (ICP) registration method incorporating incidence-angle-dependent weighting functions. By explicitly modeling the relationship between incidence angle and point cloud quality, the proposed method substantially improves registration accuracy relative to conventional ICP methods. Contribution #11 introduces an elastic fine-tuning dual recurrent framework for unsupervised non-rigid point cloud registration. The proposed method decomposed non-rigid transformations into sequential rigid transformations while enforcing elastic deformation constraints, achieving state-of-the-art performance in both rigid and non-rigid registration tasks.

Finally, large-scale sensing integration and smart construction monitoring are investigated in Contribution #10, in which an integrated monitoring platform combining point cloud data, IoT sensing, and ultra-wideband indoor positioning technologies is proposed. The developed system enabled simultaneous monitoring of construction quality and worker safety through real-time sensing and visualization, demonstrating the practical value of intelligent point cloud systems for smart construction management.

## 3. Conclusions and Future Work

The contributions in this Special Issue demonstrate the rapid advancement of intelligent point cloud processing technologies toward more accurate, robust, and efficient application-oriented perception systems. Several important future research directions also emerge from these contributions. First, the development of lightweight and efficient architectures remains essential for edge deployments. Second, multimodal fusion and cross-modal learning are expected to further improve robustness in complex and dynamic environments. Third, scalable point cloud processing frameworks capable of handling large-scale scenes remain an open challenge. Finally, trustworthy and interpretable AI-driven point cloud perception systems will become increasingly important for safety-critical applications such as autonomous systems, industrial monitoring, and robotics.

We believe that the contributions published in this Special Issue will serve as valuable references for researchers and practitioners working in point cloud processing, LiDAR sensing, robotics, and autonomous systems. We hope that they will stimulate further interdisciplinary research and foster continued innovation in intelligent 3D sensing and perception technologies.

## Data Availability

The related datasets can be found in each contribution in this Editorial.
